# Impaired Fasting Glucose in Nondiabetic Range: Is It a Marker of Cardiovascular Risk Factor Clustering?

**DOI:** 10.1155/2015/804739

**Published:** 2015-10-04

**Authors:** Giovanna Valentino, Verónica Kramer, Lorena Orellana, María José Bustamante, Cinthia Casasbellas, Marcela Adasme, Alejandra Salazar, Carlos Navarrete, Mónica Acevedo

**Affiliations:** ^1^División de Enfermedades Cardiovasculares, Facultad de Medicina, Escuela de Medicina, Pontificia Universidad Católica de Chile, 8330074 Santiago, Chile; ^2^Departamento de Matemáticas, Universidad de la Serena, La Serena, Chile

## Abstract

*Background*. Impaired fasting glucose (IFG) through the nondiabetic range (100–125 mg/dL) is not considered in the cardiovascular (CV) risk profile. *Aim*. To compare the clustering of CV risk factors (RFs) in nondiabetic subjects with normal fasting glucose (NFG) and IFG. *Material and Methods*. Cross-sectional study in 3739 nondiabetic subjects. Demographics, medical history, and CV risk factors were collected and lipid profile, fasting glucose levels (FBG), C-reactive protein (hsCRP), blood pressure (BP), anthropometric measurements, and aerobic capacity were determined. *Results*. 559 (15%) subjects had IFG: they had a higher mean age, BMI, waist circumference, non-HDL cholesterol, BP, and hsCRP (*p* < 0.0001) and lower HDL (*p* < 0.001) and aerobic capacity (*p* < 0.001). They also had a higher prevalence of hypertension (34% versus 25%; *p* < 0.001), dyslipidemia (79% versus 74%; *p* < 0.001), and obesity (29% versus 16%; *p* < 0.001) and a higher Framingham risk score (8% versus 6%; *p* < 0.001). The probability of presenting 3 or more CV RFs adjusted by age and gender was significantly higher in the top quintile of fasting glucose (≥98 mg/dL; OR = 2.02; 1.62–2.51). *Conclusions*. IFG in the nondiabetic range is associated with increased cardiovascular RF clustering.

## 1. Introduction

According to the last National Health Survey (2009), the prevalence of diabetes in Chile has increased 50% after six years (from 6.3 to 9.4%) [1, 2]. This diabetes prevalence is alarming since it is a well-known independent risk factor (RF) for cardiovascular (CV) disease. It is indeed considered a coronary risk equivalent [3, 4]. However, it has been shown that the pathophysiology of type 2 diabetes involves a slow progression from insulin resistance to alterations of fasting blood glucose (FBG). Although the latter is usually considered for the diagnosis of the metabolic syndrome (MS), it is not contemplated as an independent CV risk factor (RF) [5].

Metabolic syndrome is characterized by the clustering of several risk factors for diabetes and CV disease [5]. Insulin resistance plays a central role in the development of MS and it is associated with a higher systemic inflammation, which favors the development of atherosclerosis. It has been shown that although levels of LDL cholesterol in subjects with MS are usually normal, the LDL particles are smaller and denser than in normal subjects, which makes them more prone to oxidation and to forming atheroma [6, 7].

Despite the clear relationship between MS, diabetes, and coronary heart disease, impaired fasting blood glucose (IFG) in the nondiabetic range (100–125 mg/dL) has not been contemplated as part of the CV risk profile, nor has its prevalence been determined across the National Health Survey. Moreover, there are some scientific societies that do not believe in the concept of the metabolic syndrome. Some relevant epidemiological studies and meta-analysis, including the Framingham study, have reported a higher risk of coronary events in patients with IFG. However, the relative risk is modest and in some cases it becomes nonsignificant when adjusted for traditional cardiovascular RF such as blood pressure (BP), cholesterol, smoking, and obesity [3, 8–10].

Although current literature does not consider IFG (100–125 mg/dL) as an independent cardiovascular RF, it is important to consider that it maintains a close relationship with traditional CV RFs. Therefore, IFG could be a marker of RF clustering. This is important since it is known that clustering of CV RF has a multiplicative effect on the risk of CV disease and is associated with subclinical changes of carotid intima-media thickness (C-IMT) [7]. In addition, fasting blood glucose is an easy and commonly measured parameter in clinical practice and there are scarce data regarding its association with CV risk and RF clustering.

The main aim of this study was to compare the clustering of cardiovascular RF between nondiabetic subjects normal fasting glucose (NFG) and IFG.

## 2. Material and Methods

This was a cross-sectional study in 3739 subjects (36% women) who were evaluated in a CV prevention program in Santiago between 2002 and 2014. Exclusion criteria were subjects with a previous diagnosis of diabetes, fasting blood glucose ≥ 126 mg/dL and those in treatment with metformin. All subjects signed an informed consent, which authorized to use their data anonymously for academic purposes.

### 2.1. General Protocol

Subjects were interviewed by the nurse of the program, who collected demographic data, medical history, medication intake, and history of traditional cardiovascular RF: dyslipidemia (DLP), hypertension (HTA), smoking, physical activity, and obesity.

Subjects presented with 12-hour fasting and venous samples were taken to determine lipid profile, fasting blood glucose, and high sensitive C-reactive protein (hsCRP). Systolic blood pressure (SBP) and diastolic (DBP) blood pressure were measured on three separate occasions and the average was calculated according to the guidelines of “Seventh Report of the Joint National Committee” [11]. The estimated 10-year risk was calculated according to the Framingham score [12]. Waist circumference, body weight, and height were also measured and body mass index (BMI) was calculated. The waist was measured at the midpoint between the iliac crest and the last rib.

Finally, subjects underwent a submaximal exercise stress test in which aerobic capacity in METS was determined when subjects reached the theoretical maximum heart rate or when they were exhausted.

### 2.2. Venous Blood Samples

The lipid profile, fasting blood glucose, and hsCRP were determined in the laboratory with the following techniques:

- Total cholesterol, HDL cholesterol, and triglycerides were determined by standard enzymatic methods (Hitachi analyzer).
- LDL cholesterol was calculated by the Friedewald formula.
- Glucose was determined with the glucose oxidase method.
- hsCRP was measured in a subsample of 1618 subjects with the nephelometric method (Dade Behring).

### 2.3. Cardiovascular Risk Factors

The following criteria were used to determine CV risk factors. 

*(1) Dyslipidemia*. Subjects with a previous diagnosis of DLP with or without drug treatment and/or LDL ≥130, HDL <40 in men and <50 in women, and non-HDL cholesterol ≥ 160 mg/dL, respectively. 

*(2) Hypertension*. Subjects with a previous diagnosis of hypertension with or without drug treatment or with SBP ≥ 140 mmHg and/or DBP ≥ 90 mmHg during the study. 

*(3) Current Smoking*. Subjects who had smoked daily ≥ 1 cigarette in the last month. 

*(4) Physical Inactivity*. Subjects who did not engage in any regular physical activity at least 1 time per week. 

*(5) Obesity*. Subjects with a BMI ≥ 30 kg/m^2^.

### 2.4. Statistical Analysis

For analysis, subjects were divided into two groups according to fasting blood glucose: (1) impaired fasting blood glucose (IFG): subjects with FBG between 100 and 125 mg/dL; (2) controls with normal fasting glucose (NFG): those with FBG < 100 mg/dL. Cardiovascular RF and demographic data were compared between groups using *t*-test for independent samples. Significant differences were considered when the *p* value was <0.05.

In addition, a proportional odds model adjusted by gender and age was calculated to determine differences in the clustering of CV RF among FBG levels. Proportional odds determined the probability of presenting ≥1 CV RF and ≥3 CV RF according to fasting blood glucose level, as a continuous variable and quintiles (Quintile 1: ≤83 mg/dL; Quintile 2: 84–87 mg/dL; Quintile 3: 88–92 mg/dL; Quintile 4: 93–97 mg/dL; Quintile 5: 98–125 mg/dL).

## 3. Results

Mean age of the sample was 52 ± 13 years (36% were women); 559 (15%) subjects had IFG, 74% had DLP, 30% had hypertension, 21% were current smokers, and 52% were physically inactive. Only 43 hyperglycemic subjects (8%) presented with isolated IFG.

Table 1 compares general demographic characteristics, anthropometric data, and CV RF between NFG and IFG subjects adjusted by age and gender. IFG individuals had higher mean age, BMI, and waist circumference and a higher prevalence of DLP (79% versus 74%; *p* < 0.001), hypertension (34% versus 25%; *p* < 0.001), and obesity (29% versus 16%; *p* < 0.001) than NFG subjects. There were no significant differences in the prevalence of reported physical inactivity and smoking. However, aerobic capacity measured in METS was significantly lower in subjects with IFG compared to subjects with NFG (11 versus 12 METS, resp.; *p* = 0.01). In addition, IFG subjects had a significantly higher Framingham risk score than NFG subjects (8% versus 6%; *p* < 0.001).

Table 2 compares biochemical variables and BP levels between IFG and NFG individuals. Subjects with IFG had higher non-HDL cholesterol, SBP, DBP, and hsCRP (*p* < 0.001) and lower HDL (*p* < 0.001).

**Figure 1:**
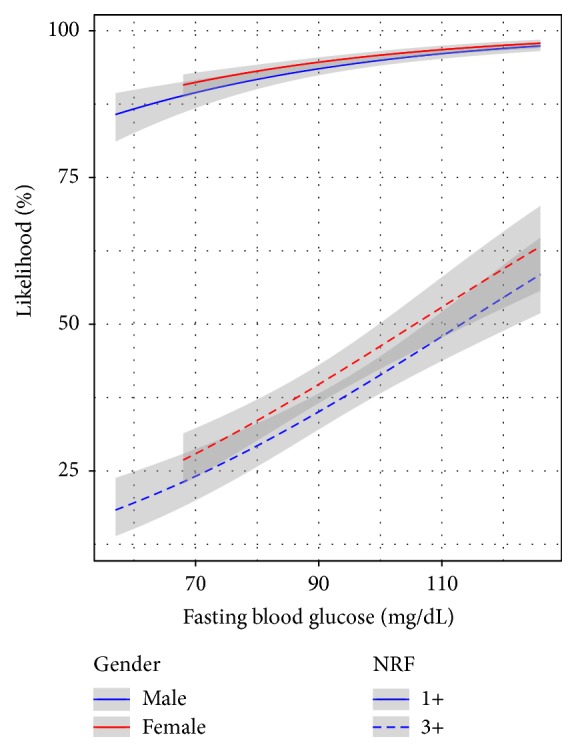
Proportional odds model for cardiovascular risk factors clustering and fasting blood glucose levels. Age (OR = 1.03; 1.02–1.03; *p* < 0.0001); gender (OR = 1.04; 0.90–1.20; *p* = NS); fasting blood glucose (OR = 1.03; 1.02–1.04; *p* < 0.0001). OR = Odds Ratio; NRF = number of risk factors; 1+ = 1 or more risk factors; 3+ = 3 or more risk factors. The figure shows the proportional odds model for determining the likelihood of presenting ≥3 and ≥1 cardiovascular risk factors according to fasting blood glucose levels in males and females adjusted by age. Per each mg/dL increase of fasting blood glucose, the probability of presenting ≥3 FR increased by 3% (OR = 1.03; 1.02–1.04; *p* < 0.001).

**Figure 2:**
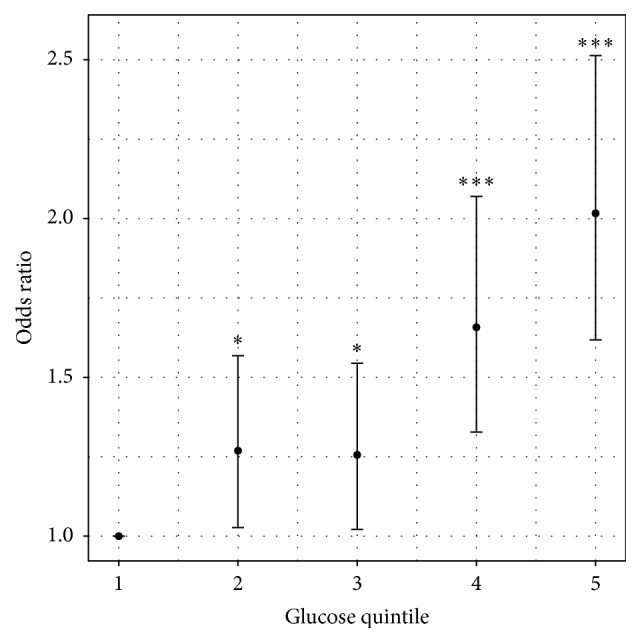
Likelihood of presenting 3 or more cardiovascular risk factors according to fasting blood glucose quintile adjusted by age and gender. ^*∗*^ *p* < 0.05; ^*∗∗∗*^ *p* < 0.0001. Fasting blood glucose quintiles: Quintile 1: ≤83 mg/dL; Quintile 2: 84–87 mg/dL; Quintile 3: 88–92 mg/dL; Quintile 4: 93–97 mg/dL; Quintile 5: 98–125 mg/dL.

Finally, subjects with IFG had a higher mean CV RF load compared to NFG subjects, in both men (2.2 versus 1.9; *p* < 0.001) and women (2.5 versus 1.9; *p* < 0.001). According to the proportional odds model, the best determinants of RF clustering were age and FBG (OR = 1.03; *p* < 0.001 for both). There were no significant differences between genders (Figure 1). In addition, the probability (OR) of having 3 or more CV RF increased significantly when glucose quintiles increased. Odds ratio was twofold higher in the top quintile (FBG ≥ 98 mg/dL) compared with the bottom quintile (≤83 mg/dL) of FBG as shown in Figure 2.

## 4. Discussion

This study demonstrated that IFG was associated with worse biochemical parameters, higher BP levels, and a higher prevalence of traditional cardiovascular RF. Also IFG was associated with some nontraditional CV risk factors, such as higher hsCRP levels and a lower aerobic capacity measured in METS. Therefore, we demonstrated that FBG is a marker of clustering of CV RF, even at levels lower than 100 mg/dL.

Epidemiological studies have reported that IFG, glucose intolerance, and high glycosylated hemoglobin in nondiabetic subjects are associated with an increased risk of coronary and vascular events [3, 8–10, 13, 14]. However, these associations tend to decrease when adjusted for traditional cardiovascular RF such as BP and cholesterol. In these studies, postload glucose is the only marker that has prevailed as an independent, but weak, cardiovascular RF when adjusted by traditional RF [8, 14]. The results of the present study, in which FBG was associated directly and significantly with the clustering of CV RF, may explain the relationship described previously between IFG and CV risk.

Henry et al. in 2002 reported that IFG only was a predictor of CV mortality in subjects with blood pressure ≥ 140/90, that is, with hypertension, while in normotensives it was not associated with a higher CV risk [9]. This reinforces the concept of controlling conventional CV RF over FBG. However, the same study reported that cholesterol, triglycerides, and BMI were not good predictors of CV mortality in subjects with IFG as they were in subjects with NFG. In that study, CV mortality was significantly higher when hypertension and IFG appeared together, but it was not higher when one of them appeared alone. This highlights the importance of RF clustering in producing risk.

The prevalence of IFG alone, that is, without the presence of other RFs, only was 8%. This finding underscores the concept of aggregation of risk markers. As an example here, the metabolic syndrome is a very good representative, in which different risk parameters such as IFG (and not necessarily RF) work together increasing future CV and metabolic risk. In our study, IFG subjects presented with worse parameters: lower HDL, higher triglycerides, higher BP levels, and higher waist circumference. Furthermore, IFG subjects had higher hsCRP levels than subjects with NFG. Today, hsCRP is accepted as a CV and metabolic risk factor. This is relevant in clinical practice, because both IFG and NFG subjects present with similar LDL cholesterol levels, which could be interpreted by clinicians as if these subjects would have the same CV risk, if fasting glucose levels were not considered in the risk equation.

In 2002, Hoogwerf et al. reported that fasting glucose quintiles throughout the nondiabetic range were graded and continuously associated with cardiovascular RF [15]. They also assessed the prevalence of coronary heart disease (CHD) through glucose quintiles and found a direct and significant association, even when adjusting for traditional and nontraditional RF. They found that the upper 3 blood glucose quintiles (fasting blood glucose >86 and <125 mg/dL) gave an OR of 1.73, 2.19, and 2.50 for CHD, which suggested that FBG could be an independent CV risk factor.

Recently, the American Heart Association (AHA) has described the concept of “ideal cardiovascular health,” in which subjects might meet criteria for both, a healthy lifestyle (not smoking, healthy diet, normal weight, and physical activity) and optimal values for biochemical parameters (BP, cholesterol, and FBG) [16]. Within this concept, those subjects that could be considered healthy by not having traditional RF such as hypertension, diabetes, or obesity, but still having IFG, prehypertension, or overweight would lose score for “ideal CV health.” In clinical practice, this means that the prevention of CV disease should focus not only on the control of traditional CV RF when they are already present, but also on preventing their occurrence. In this regard, along with the weight and blood pressure, FBG is an easy-to-measure parameter to detect CV RF clustering, even with glucose levels within the “optimal” range.

The last Chilean National Health Survey revealed a median fasting blood glucose of 98 mg/dL, which means that 50% of the Chilean population had a FBG value ≥ 98 mg/dL that despite being in an “optimal range” would be associated with a double chance of having 3 or more cardiovascular RFs according to our present study when compared to subjects with FBG ≤ 83 mg/dL [1]. Looking at this survey, the data are worrisome, as from age 45 and older an increase is observed in mean FBG to values above 100 mg/dL. In agreement with these observations, the prevalence of metabolic syndrome in Chile is 35% and the prevalence of diabetes increased dramatically between 2003 and 2009 [1, 17]. Therefore, it is of clinical relevance for the years to come that clinicians should be aware that increases in FBG should be taken into account as early markers of clustering of RF. In this regard, it has been widely demonstrated that intensive interventions on diet and exercise are very effective treatments for glycemic control and prevention of diabetes in these patients [18]. It is important here to highlight that in our population even though both groups, IFG and NFG subjects, had fairly good functional capacity determined by METs, there was a clear better performance in NFG group compared to IFG group. Aerobic capacity, which is closely related to physical activity, has been reported as a predictor of CV events [19, 20]. It has been reported that each MET of higher aerobic capacity reduces the risk of CV mortality by 15% [19]. Therefore, reinforcing lifestyle changes in subjects with IFG should be widely applied in the clinical practice.

In summary, IFG is a marker of increased cardiovascular RF clustering in nondiabetic subjects even through normal FBG ranges (<100 mg/dL). This study emphasizes the importance of FBG as a factor to consider in the prevention of overall cardiovascular risk.

This study has limitations. First, it is not free of selection biases as subjects were those who voluntarily attended a primary CV prevention program. Second, insulin resistance was not measured although it has been associated with MS pathophysiology. However, in the last definition of MS (ATP III harmonized) it has not been included. Neither was glucose tolerance measured. Postload glucose levels have shown a better association with CV risk than IFG [9, 15]. However, this study sought to demonstrate whether FBG would be a good marker of CV RF clustering, mainly because it has a low cost and is commonly measured in the clinical setting. Finally, the main limitation was that, as an observational study, no causality could be reported.

## Figures and Tables

**Table 1:** Mean values for general demographics, anthropometric variables, aerobic capacity, and cardiovascular risk factors in subjects with normal and impaired fasting blood glucose adjusted by age and gender.

	NFG (FBG <100 mg/dL) (*n* = 3180)	IFG (FBG = 100–125 mg/dL) (*n* = 559)	*p*
Age (years)	51 ± 13	58 ± 11	<0.001
Female gender (%)	38%	24%	<0.001
Anthropometric variables			
Waist circumference (cm)	91 ± 10	95 ± 11	<0.001
BMI (Kg/m^2^)	26.6 ± 4	28.2 ± 4	<0.001
Aerobic capacity (METS)	12 ± 3	11 ± 3	<0.001
Framingham score (%)	6 ± 5	8 ± 5	<0.001
Cardiovascular risk factors			
Obesity (%)	16%	29%	<0.001
Hypertension (%)	25%	34%	<0.001
Dyslipidemia (%)	74%	79%	0.05
Smoking (%)	20%	21%	NS
Physical inactivity (%)	51%	55%	NS

Data expressed in mean ± SD except where indicated.

NFG = normal fasting glucose; IFG = impaired fasting glucose; FBG = fasting blood glucose; BMI = body mass index; SD = standard deviation.

**Table 2:** Mean values for biochemical variables and blood pressure in subjects with normal and impaired fasting blood glucose.

	NFG (FBG <100 mg/dL) (*n* = 3180)	IFG (FBG = 100–125 mg/dL) (*n* = 559)	*p*
FBG (mg/dL)	88 ± 6	106 ± 6	<0.001
Total cholesterol (mg/dL)	206 ± 40	211 ± 41	0.03
Triglycerides (mg/dL)	137 ± 111	167 ± 112	<0.001
HDL cholesterol (mg/dL)	54 ± 15	50 ± 13	<0.001
LDL cholesterol (mg/dL)	126 ± 35	128 ± 37	NS
Non-HDL cholesterol (mg/dL)	153 ± 40	161 ± 41	<0.001
Systolic blood pressure (mmHg)	122 ± 14	130 ± 15	<0.001
Diastolic blood pressure (mmHg)	76 ± 8	80 ± 8	<0.001
hsCRP (mg/L)	1.8 ± 1.9	2.2 ± 2.1	<0.01

Data expressed in mean ± SD.

NFG = normal fasting glucose; IFG = impaired fasting glucose; FBG = fasting blood glucose; HDL = high density lipoprotein; LDL = low density lipoprotein; hsCRP = high sensitive C-reactive protein; SD = standard deviation.

## References

[1] MINSAL (2009). *Segunda Encuesta Nacional de Salud*.

[2] MINSAL Primera Encuesta Nacional de Salud. http://epi.minsal.cl/epi/html/invest/ENS/InformeFinalENS.pdf.

[3] Sarwar N., Gao P., Seshasai S. R. (2010). Diabetes mellitus, fasting blood glucose concentration, and risk of vascular disease: a collaborative meta-analysis of 102 prospective studies. *The Lancet*.

[4] Schramm T. K., Gislason G. H., Køber L. (2008). Diabetes patients requiring glucose-lowering therapy and nondiabetics with a prior myocardial infarction carry the same cardiovascular risk: a population study of 3.3 million people. *Circulation*.

[5] Alberti K. G. M. M., Eckel R. H., Grundy S. M. (2009). Harmonizing the metabolic syndrome: a joint interim statement of the International Diabetes Federation Task Force on Epidemiology and Prevention; National Heart, Lung, and Blood Institute; American Heart Association; World Heart Federation; International Atherosclerosis Society; and International Association for the Study of Obesity. *Circulation*.

[6] Gerber P. A., Thalhammer C., Schmied C. (2013). Small, dense LDL particles predict changes in intima media thickness and insulin resistance in men with type 2 diabetes and prediabetes—a prospective cohort study. *PLoS ONE*.

[7] Acevedo M., Arnaíz P., Corbalán R. (2009). Modificación del grosor intima-media carotídeo según factores de riesgo clásicos y síndrome metabólico con o sin inflamación. *Revista Chilena de Cardiología*.

[8] Meigs J. B., Nathan D. M., D'Agostino R. B., Wilson P. W. F. (2002). Fasting and postchallenge glycemia and cardiovascular disease risk: the framingham offspring study. *Diabetes Care*.

[9] Henry P., Thomas F., Benetos A., Guize L. (2002). Impaired fasting glucose, blood pressure and cardiovascular disease mortality. *Hypertension*.

[10] Levitan E. B., Song Y., Ford E. S., Liu S. (2004). Is nondiabetic hyperglycemia a risk factor for cardiovascular disease? A meta-analysis of prospective studies. *Archives of Internal Medicine*.

[11] Chobanian A. V., Bakris G. L., Black H. R. (2003). The seventh report of the joint national committee on prevention, detection, evaluation, and treatment of high blood pressure: the JNC 7 report. *The Journal of the American Medical Association*.

[12] Wilson P. W. F., D'Agostino R. B., Levy D., Belanger A. M., Silbershatz H., Kannel W. B. (1998). Prediction of coronary heart disease using risk factor categories. *Circulation*.

[13] Selvin E., Lazo M., Chen Y. (2014). Diabetes mellitus, prediabetes, and incidence of subclinical myocardial damage. *Circulation*.

[14] Smith N. L., Barzilay J. I., Shaffer D. (2002). Fasting and 2-hour postchallenge serum glucose measures and risk of incident cardiovascular events in the elderly: the Cardiovascular Health Study. *Archives of Internal Medicine*.

[15] Hoogwerf B. J., Sprecher D. L., Pearce G. L. (2002). Blood glucose concentrations ≤125 mg/dl and coronary heart disease risk. *The American Journal of Cardiology*.

[16] Folsom A. R., Yatsuya H., Nettleton J. A., Lutsey P. L., Cushman M., Rosamond W. D. (2011). Community prevalence of ideal cardiovascular health, by the American Heart Association definition, and relationship with cardiovascular disease incidence. *Journal of the American College of Cardiology*.

[17] Valenzuela B. A. A., Maíz A., Margozzini P. (2010). Prevalencia de síndrome metabólico en población adulta Chilena: Datos de la Encuesta Nacional de Salud 2003. *Revista Médica de Chile*.

[18] Knowler W. C., Barrett-Connor E., Fowler S. E. (2002). Reduction in the incidence of type 2 diabetes with lifestyle intervention or metformin. *The New England Journal of Medicine*.

[19] Kodama S., Saito K., Tanaka S. (2009). Cardiorespiratory fitness as a quantitative predictor of all-cause mortality and cardiovascular events in healthy men and women: a meta-analysis. *Journal of the American Medical Association*.

[20] Barry V. W., Baruth M., Beets M. W., Durstine J. L., Liu J., Blair S. N. (2014). Fitness vs. fatness on all-cause mortality: a meta-analysis. *Progress in Cardiovascular Diseases*.

